# Shark fisheries in the Southeast Pacific: A 61-year analysis from Peru

**DOI:** 10.12688/f1000research.4412.2

**Published:** 2016-04-12

**Authors:** Adriana Gonzalez-Pestana, Carlos Kouri J., Ximena Velez-Zuazo

**Affiliations:** 1 Departamento de Biología Marina y Econegocios, Universidad Cientifica del Sur, Lima, Peru; 2 ecOceánica, Lima, Peru; 3 Department of Biology, University of Puerto Rico, San Juan, Puerto Rico

**Keywords:** endangered species, fish, elasmobranchs, fishing, landing reports, ocean, coastal, sustainable management, Peru, southeast Pacific

## Abstract

Peruvian waters exhibit high conservation value for sharks. This contrasts with a lag in initiatives for their management and a lack of studies about their biology, ecology and fishery. We investigated the dynamics of Peruvian shark fishery and its legal framework identifying information gaps for recommending actions to improve management. Further, we investigated the importance of the Peruvian shark fishery from a regional perspective. From 1950 to 2010, 372,015 tons of sharks were landed in Peru. From 1950 to 1969, we detected a significant increase in landings; but from 2000 to 2011 there was a significant decrease in landings, estimated at 3.5% per year. Six species represented 94% of landings: blue shark ( *Prionace glauca*), shortfin mako ( *Isurus oxyrinchus*), smooth hammerhead ( *Sphyrna zygaena*), common thresher ( *Alopias vulpinus*), smooth-hound ( *Mustelus whitneyi*) and angel shark ( *Squatina californica*). Of these, the angel shark exhibits a strong and significant decrease in landings: 18.9% per year from 2000 to 2010. Peru reports the highest accumulated historical landings in the Pacific Ocean; but its contribution to annual landings has decreased since 1968. Still, Peru is among the top 12 countries exporting shark fins to the Hong Kong market. Although the government collects total weight by species, the number of specimens landed as well as population parameters (e.g. sex, size and weight) are not reported. Further, for some genera, species-level identification is deficient and so overestimates the biomass landed by species and underestimates the species diversity. Recently, regional efforts to regulate shark fishery have been implemented to support the conservation of sharks but in Peru work remains to be done.

## Introduction

Overexploitation and bycatch imperils sharks ( Baum *et al.*, 2003; Camhi *et al.*, 2008; Ferretti *et al.*, 2008; Ward & Myers, 2005). Sharks have been commercially fished for 200 years ( Kroese & Sauer, 1998), but since the 1980’s its fishery has rocketed. The major driver has been a growing demand for shark fins, which can cost up to 1000 Euros per fin ( Oceana, 2010). However, sharks are vulnerable to overfishing due to their life-history characteristics including slow growth, late maturity and small litter size ( Musick, 1999). Their jeopardized situation is worsened by major gaps of knowledge that hinder the design and implementation of conservation and management actions. These gaps include a limited understanding of shark fishery characteristics (e.g. captures, gear, fishing areas, seasons; Bonfil, 1994; Rose, 1996), species diversity and the dynamics of their populations ( Camhi *et al.*, 2008; Smale, 2008).

Within the Pacific Ocean more than half of the reported landings are from the western and central Pacific (e.g. Japan, Hawaii, Camhi *et al.*, 2008). Fishing vessels in the eastern Pacific also target sharks, but their contribution to the total Pacific catch and their impact on sharks is poorly known due to a lack of detailed landing reports. Three countries in the eastern Pacific; Costa Rica, Ecuador, and Peru, are important suppliers of shark fins for the Asian market, the major consumer of shark fins in the world ( Oceana, 2010). Shark fishery information is available for Costa Rica and Ecuador either through published papers (i.e. Carr *et al.*, 2013; Jacquet *et al.*, 2008; Schiller *et al.*, 2015) or by mass-media coverage (e.g. the movie Sharkwater), but for Peru the information is scarce. Yet, the coast of Peru exhibits a high degree of shark species richness and functional richness while the high seas (i.e. international waters) off Peru have a high value for shark conservation ( Lucifora *et al.*, 2011). In light of this, an understanding of the dimension and dynamics of shark fishery in Peru and its relative contribution to total landings from the Pacific is crucial to establish both local and regional management actions.

In this study, the past dynamics and current status of Peruvian shark fishery were investigated. Unpublished data, governmental reports and published literature were compiled to establish a baseline of information about sharks in Peru. This study aimed to (1) describe and analyze the Peruvian shark fishery, (2) identify and analyze the Peruvian shark fishery contribution in the Pacific basin, (3) analyze the international commerce of shark fins and meat in Peru (4) describe and analyze the conservation status of sharks in Peru and its legal framework (national and international), and (5) identify the current gaps in information and regulation hindering management actions in order to offer recommendations for improving management and conservation. This information would enhance local and regional management actions and would promote research in shark fisheries management. This study represents, so far, the first comprehensive investigation of Peruvian shark fishery research.

## Methods

For describing and analyzing the Peruvian shark fishery, we combined total landing information (tons of sharks - t) from FAO (Food and Agriculture Organization of the United Nations, 1950–1963) and IMARPE (Instituto del Mar del Perú, 1964–2010). FAO reports landings by country using different alternatives for classification. For this study, we used reports using the ASFIS system. IMARPE records the landings of Peruvian small-scale fishery which according to the Peruvian fisheries regulations, is defined as boats with a maximum capacity of 32.6m ^3^ Gross Registered Tonnage (GRT), up to 15 meters of length and operate predominantly using manual work ( El Peruano, 2001). IMARPE, until 1996, reported landings of sharks without making any distinction regarding species but dividing them in three main groups which we pooled together: "tiburon" (sharks), “toyos” (smoothhounds *Mustelus* sp.), and “angelotes” (angel sharks, *Squatina* sp.). From 1996, landing reports are presented at the species level. To determine the six most landed shark species in Peru, we gathered species-specific landing information, from 1996 to 2010, published in the annual fishing reports of IMARPE ( Estrella-Arellano & Guevara-Carrasco, 1998; Estrella *et al.*, 1998; Estrella-Arellano *et al.*, 1999a; Estrella-Arellano *et al.*, 1999b; Estrella-Arellano *et al.*, 2000a; Estrella-Arellano *et al.*, 2000b; Estrella-Arellano *et al.*, 2001; Flores *et al.*, 1994; Flores *et al.*, 1997; Flores *et al.*, 1998a; Flores *et al.*, 1998b; Flores *et al.*, 2001).

To determine the locations with the highest shark landings, we used information from IMARPE between 1996 and 2010; to determine the fishing method used to catch sharks, we used information from IMARPE between 1996 and 2000. To create a map of Peru with landing points along the coast, we used Maptool a resource available from SEATURTLE.ORG.

To investigate the trend and change in shark landings in Peru between 1950 and 2010, we used a generalized least squares (GLS) to fit a linear model, maximizing the restricted log-likelihood (REML), with unequal variances to account for measurement uncertainty. For this, we used the package *nlme* ( Pinheiro *et al.*, 2014) implemented in R 2.13.2. To estimate the confidence intervals (CI) of the GLS model parameters we used a nonparametric bootstrapping with replacement (R=1000) of the resulting coefficients using the package boot ( Canty & Ripley, 2013; Davison & Hinkley, 1997) in R 2.13.2. We established a time-scale length of 10 years to maximize detection of any significant trend in landings over this period of time. The decision for using this length was guided by results from preliminary tests, where we observed significance (p-value <0.05) over this time-scale compared to larger or smaller scales that were mostly non-significant. We partitioned the data every ten years, except for the last segment, from 2000–2010, where we included 11 years to use all the data. We used this same time-scale segment (2000 to 2010) to investigate the trend in landings by species.

We estimated the average catch per unit effort (CPUE) for the most commonly landed shark species between 2002 and 2007. According to Alfaro-Shigueto *et al.* (2010), the fishing effort of the small scale fishery that uses gillnets is 100 000 km of nets per annum and for the longline the effort is 80 million hooks set per annum. We extrapolated these numbers to the Peruvian small-scale shark fishery so we can obtain an estimated CPUE. First for each of the most landed species, we calculated the landings per year using a particular fishing gear (gillnet or hooks). Second, we calculated the CPUE (per species, year and fishing gear): shark species biomass per 100 m per year and shark species biomass per 100 hooks per year. Finally, we obtained an average CPUE for the years between 2002 and 2007.

To investigate the correspondence between the six most landed shark species and El Nino Southern Oscillation (ENSO), we used a linear regression analysis, which combined monthly landings by species with monthly values of the Multivariate ENSO Index (MEI). MEI is the value of the first unrotated Principal Component of the integrated analysis of six variables: sea surface temperature, sea-level pressure, surface wind, surface air temperature, and total cloudiness fraction of the sky ( http://www.esrl.noaa.gov/psd/enso/mei/). For this analysis, we implemented the linear regression function (lm) in R 3.02 in RStudio 0.98.501 ( www.rstudio.org).

We identified and analyzed the contribution of the Peruvian shark fishery to the Pacific basin by comparing total landings of chondrichthyans (sharks, batoids and chimaeras) followed by sharks only (i.e. using ASFIS system, www.fao.org) with landings reported by other countries fishing in the Pacific Ocean. When this information was filtered for sharks only, we were left with 24 countries reporting at this level. For the analysis, we compiled the statistics reported by country to FAO, between 1950 and 2010, using the software FishStatJ v.2.0.0 ( http://www.fao.org/fishery/statistics/software/en).

For estimating and analyzing the annual and spatial dynamics of shark fin and meat international commerce, we obtained the tons imported and exported, by year and by country, and the free-on-board amount in American dollars ($ FOB) from 1997 to 2012, reported by commodity code by the Superintendencia Nacional de Aduanas y Administracion Tributarias (SUNAT, www.aduanet.gob.pe). Here, we used the same analytical approach used to investigate the trend and change in shark landings over a period of time to test for significant changes in the export and import of shark fins. For this analysis, the difference was that the time-scale length investigated was of five years, except for the period from 2007 to 2012 that was of six years.

Finally, we described and analyzed the conservation status of landed sharks in Peru using the information from Red List of the International Union of the Conservation of Nature (IUCN, www.redlist.org, accessed in March 2012). To determine shark resilience, we used the global online database FishBase ( www.fishbase.org, accessed in May, 2012). For describing and analyzing legal framework at the national level, we used information from Ministerial Resolution (RM) Nº 209-2001-PE, which regulates Peruvian shark fishery. At the international level, we used information from The Convention on International Trade in Endangered Species of Wild Fauna and Flora (CITES, www.cites.org, accessed in March 2013); Convention on Migratory Species (CMS, www.cms.int, April 2012); and United Nations Convention on the Law of the Sea (UNCLOS). For all the analyses and graphic representation of data, we used R 2.13.2 ( R Development Core Team, 2011).

## Results

### National perspective of the Peruvian shark fishery

**Figure 1.**
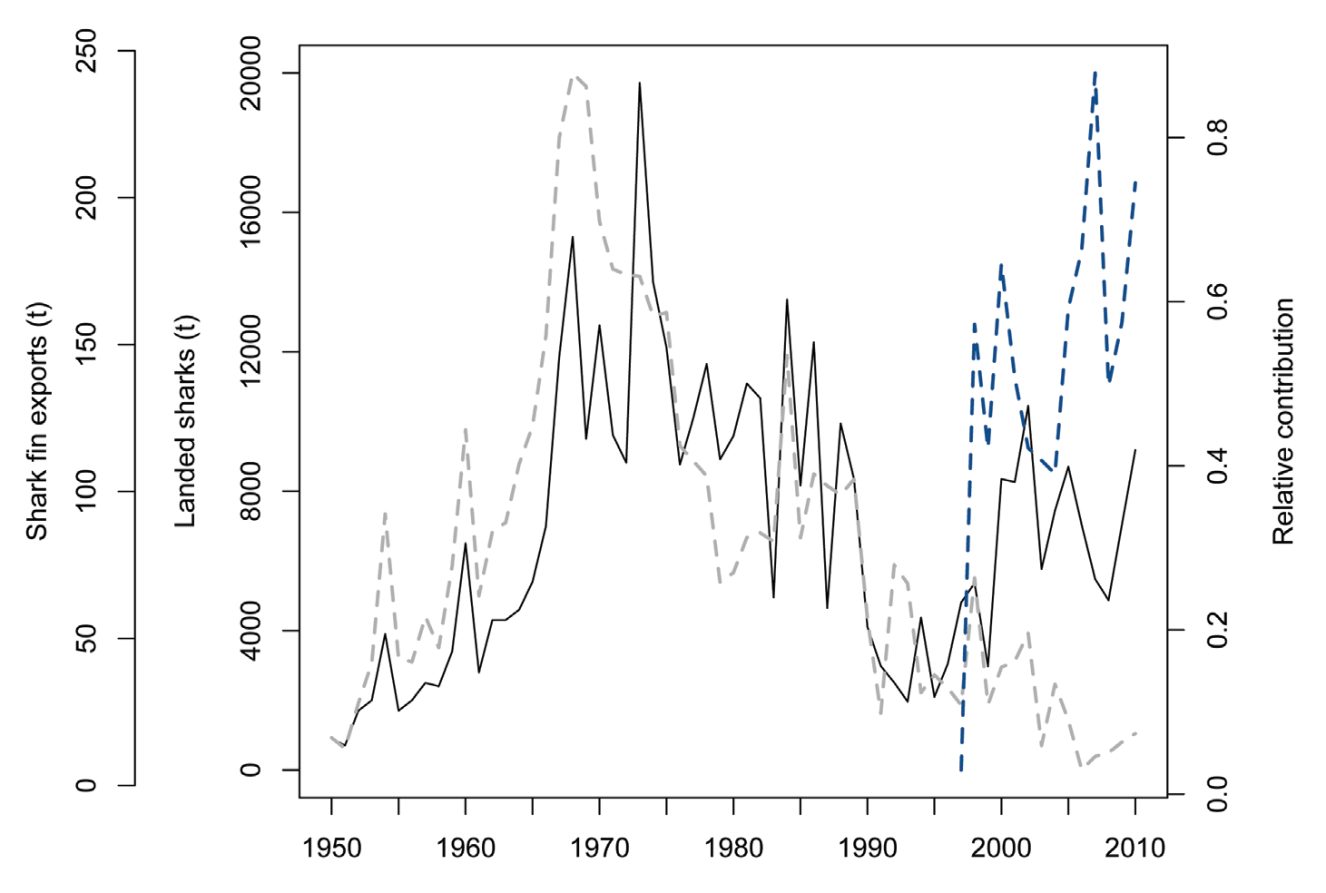
Temporal dynamics of shark landings and international trade in Peru and regional contribution.

Between 1950 and 2010, the Peruvian small-scale Peruvian fishery landed 372,015 t of sharks with a landing average of 6,099 t per year (SD ± 4251.3), a minimum of 700 t in 1951 and a maximum landing of 19,718 t in 1973 ( Figure 1). Landings fluctuated over time exhibiting an increase between 1950 and 1973 with significant increases from 1950 to 1959 (slope=0.136, 95% CI: 0.057, 0.242, Table 2), which corresponds to an increase of 14.6% per year, and from 1960 to 1969 (slope=0.133, 95% CI: 0.041, 0.228), which corresponds to an increase of 14.2% per year. From 1973, landings decreased to a minimum of 1961 t in 1993. During the last 11 years of landings, from 2000 to 2010, a significant decrease in landings was detected (slope=-0.035, 95% CI: -0.0515, -0.0039), which corresponds to a decline of 3.45% per year.

Shark landings in Peru from 1950 to 2010 (solid line), its relative contribution to landings reported for the Pacific Ocean (dotted line), and shark fin exports from 1997 to 2010 (blue dotted line).

In Peruvian waters, 60 species of sharks are reported ( Chirichigno, 1978; Cornejo & Chirichigno, 2012; Nakaya *et al.*, 2009). Of these, 33 species interact with the Peruvian small-scale fishery ( Bustamante, 1997; Romero & Bustamante, 2007), but landing statistics were limited to 18 species ( Table 1). Six species comprise the majority of the shark fishery: blue shark ( *Prionace glauca*), shortfin mako shark ( *Isurus oxyrinchus*), smooth hammerhead ( *Sphyrna zygaena*), smooth-hound shark ( *Mustelus whitneyi*), common thresher ( *Alopias vulpinus*), and the angel shark ( *Squatina californica*). From 1996 to 2010, they represented 98% of total shark landings. Blue shark is the most common species landed in Peru (42% of shark landings), followed by shortfin mako (20%), smooth hammerhead (15%), smooth-hound shark (7%), common thresher (6%), and angel shark (4%). Since the shark fishery is dominated by these six species, their temporal and spatial patterns were analyzed. The other 12 shark species landed represented altogether 2% of the total landings from 1996 to 2010 (see recorded landing species in Table 1).

**Table 1.** Summarized biological information, conservation status (CS) and international and national legal regime for the sharks reported in Peruvian fishery. IUCN: International Union for the Conservation of Nature, CITES: Convention on International Trade in Endangered Species, CMS: Convention on Migratory Specie, UNCLOS: United Nations Convention on the Law of the Sea.

Specie	Common name	Landings recorded	CS		Legal Regime				Size at maturity (cm)		Resilience ^b^
			IUCN ^a^		International			National			
			Status	Pop. Trend	CITES	CSM	UNCLOS	Minimum length at capture (cm)	Female	Male	
**Fam. Squatinidae**											
*Squatina* *californica*	Pacific angelshark	X	NT	UNK					90–100 ( Natanson & Cailliet, 1986)		Very low
**Fam. Scyliorhinidae**											
*Schroederichthys* *chilensis*	Redspotted catshark	X	DD	UNK					52–54 ( Lamilla, 2004)	42–46 ( Lamilla, 2004)	Low
**Familia Alopiidae**											
*Alopias* *superciliosus*	Bigeye thresher		VU	DEC			X		332–355 ( Chen *et al.*, 1997)	270–300 ( Chen *et al.*, 1997)	Very low
*Alopias pelagicus*	Pelagic thresher		VU	DEC			X		282–292 ( Liu *et al.*, 1999)	267–276 ( Liu *et al.*, 1999)	Very low
*Alopias vulpinus*	Common Thresher	X	VU	DEC			X		303 ( Cailliet *et al.*, 1983)	293–311 ( Cailliet *et al.*, 1983)	Very low
**Fam. Carcharhinidae**											
*Carcharhinus* *brachyurus*	Copper shark	X	NT	UNK				150	227–244 ( Walter & Ebert, 1991)	206–235 ( Walter & Ebert, 1991)	Very low
*Carcharhinus* *falciformis*	Silky shark	NT	DEC			X		150	193–200 ( Oshitani *et al.*, 2003)	186 ( Oshitani *et al.*, 2003)	Very low
*Carcharhinus* *galapagensis*	Galapagos shark		NT	UNK				150	215–250 ( Wetherbee *et al.*, 1996)	205–250 ( Wetherbee *et al.*, 1996)	Very low
*Carcharhinus* *limbatus*	Blacktip shark		NT	UNK				150	150–156 ( Killam, 1987)	130–145 ( Killam, 1987)	Low
*Carcharhinus* *porosus*	Smalltail shark	X	DD	UNK				150	72–78 ( Compagno *et al.*, 2005)	84 ( Compagno *et al.*, 2005)	Very low
*Carcharhinus* *longimanus*	Oceanic whitetip shark		VU	DEC	II		X	150	170–190 ( Seki *et al.*, 1998)	96–170 ( Seki *et al.*, 1998)	Very low
*Carcharhinus* *leucas*	Bull shark		NT	UNK				150	180–230 ( Compagno, 1984)	157–226 ( Compagno, 1984)	Very low
*Carcharhinus* *altimus*	Bignose shark		DD	UNK				150	226 ( Compagno, 1984)	216 ( Compagno, 1984)	Very low
*Prionace glauca*	Blue shark	X	NT	UNK			X	160	170–190 ( Francis & Duffy, 2005)	190–195 ( Francis & Duffy, 2005)	Very low
*Nasolamia velox*	Whitenose shark	X	DD	UNK						140 ( Compagno, 1984)	Very low
*Rhizoprionodon* *longurio*	Pacific sharpnose shark		DD	UNK					83 ( Castillo, 1990)	93 ( Castillo, 1990)	Low
*Galeocerdo* *cuvier*	Tiger shark	X	NT	UNK					250–350 ( Randall, 1992)	226–290 ( Randall, 1992)	Low
**Familia Echinorhinidae**											
*Echinorhinus* *cookei*	Prickly shark		NT	UNK					250–300 ( Compagno, 1984)	180–200 ( Compagno, 1984)	Low
**Fam. Hexanchidae**											
*Notorynchus* *cepedianus*	Broadnose sevengill shark	X	DD	UNK					220 ( Peres & Vooren, 1991)	150 ( van Dykhuizen & Mollet, 1992)	Very low
**Fam. Lamnidae**											
*Isurus oxyrinchus*	Shortfin mako	X	VU	DEC		II	X	170	265–280 ( Stevens, 1983)	195 ( Stevens, 1983)	Very low
*Lamna nasus*	Porbeagle	X	VU	DEC	II	II	X		195 ( Francis & Duffy, 2005)	165 ( Francis & Duffy, 2005)	Very low
**Fam. Triakidae**											
*Mustelus mento*	Speckled smooth- hound	X	NT	DEC				60	86–90 ( Compagno *et al.*, 2005)	65–76 ( Compagno *et al.*, 2005)	Very low
*Mustelus whitneyi*	Humpback smooth- hound	X	VU	DEC				60	74–87 ( Compagno, 2007)	68 ( Compagno, 2007)	Very low
*Mustelus dorsalis*	Sharptooth smooth- hound	X	DD	UNK					43 ( Peres & Vooren, 1991)		Very low
*Galeorhinus* *galeus*	Tope shark	X	VU	DEC					134–140 ( Peres & Vooren, 1991)	120–135 ( Peres & Vooren, 1991)	Very low
*Triakis maculata*	Spotted houndshark	X	VU	DEC				60			Low
**Fam. Rhincodontidae**											
*Rhincodon typus*	Whale shark	X	VU	DEC	II	I					Very low
**Fam. Spyrnidae**											
*Sphyrna lewini*	Scalloped hammerhead		EN	UNK	II				210–250 ( Chen *et al.*, 1988)	140–198 ( Chen *et al.*, 1988)	Low
*Sphyrna zygaena*	Smooth hammerhead	X	VU	DEC	II				265 ( Stevens, 1984)	250–260 ( Stevens, 1984)	Low
*Sphyrna* *mokarran*	Great hammerhead		EN	DEC	II				250–300 ( Compagno, 2007)	234–269 ( Compagno, 2007)	Low
*Sphyrna tiburo*	Bonnethead		LC	UNK					80–95 ( Compagno, 1984)	68–85 ( Compagno, 1984)	Very low
**Fam. Heterodontidae**											
Heterodontus quoyi	Galapagos bullhead shark	X	DD	UNK							Low

(a) Species status: EN-Endangered, VU-Vulnerable, NT-Near Threatened, LC-Least Concern and DD-Data Deficient; Population trend: DC-Decreasing, ST-Stable, UNK-Unknown, after IUCN Red List ( www.redlist.org).

(b) Resilience: Very low: Population doubling only about every 14 years, Low: minimum population doubling 4.5–14 years, from FishBase ( www.fishbase.org).

**Figure 2.**
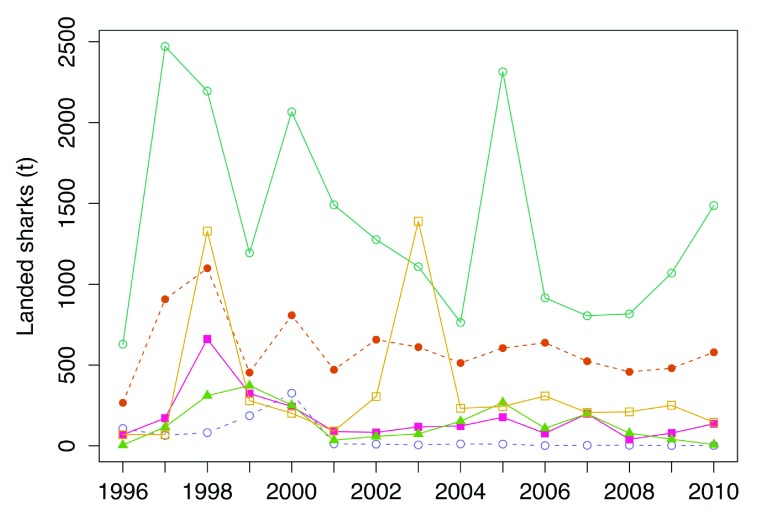
Temporal dynamics of shark landings by species. Fifteen years of annual landings for the six most important commercial shark species: blue shark- *Prionace glauca* (green solid line-open circle), mako shark- *Isurus oxyrinchus* (orange dashed line-closed circle), angel shark- *Squatina californica* (light purple dashed line-open circle), smooth-hound- *Mustelus whitneyi* (fucsia solid line-closed square), common thresher shark- *Alopias vulpinus* (light green solid line-closed triangle), and smooth hammerhead shark- *Sphyrna zygaena* (yellow solid line-open square).

Landings by species varied between 1996 and 2010 exhibiting contrasting patterns for some species and a steady decline for others ( Figure 2). Between 2000 and 2010, the landing of smooth hammerheads exhibited a significant increase (slope=0.069, 95% CI: -0.0075, 0.1516), corresponding to an increase of 7.14% per year, while significant declines in landings were detected for the shortfin mako (slope=-0.032, 95% CI: -0.0621, -0.0142) and angel shark (slope=-0.209, 95% CI: -0.3285, -0.1407). The declines of these two species were estimated at 3.16% and 18.88% per year, respectively ( Table 2). Landings of blue sharks were particularly high in 1997 and 2001, while landings of the smooth hammerhead shark were the highest in 1998 and 2003. Similar to the blue shark, landings of the mako shark exhibited an increase, albeit not as strong, in 2000. For the angel shark, an increase in landings in 2001 (3,75.3 t) was observed, followed by a steady and significant decline only interrupted by a small peak of landings in 2007 (1,51.3 t). A weak, albeit significant relationship, between monthly shark landings and MEI values for four of the six most landed species was found (blue shark, correlation coefficient-r=0.28, slope=20462, ±SEM=5443.27, p-value<0.001; shortfin mako, r=0.29, slope=11579, ±SEM=2897.78, p-value<0.001; smooth hammerhead, r=0.29, slope=12834, ±SEM=3241.57, p-value<0.001; smooth-hound, r=0.21, slope=-4317, ±SEM=1540.74, p-value<0.05).

**Table 2.** Trend and change of shark landings in Peru estimated using generalized least squares models for all sharks landed from 1950 to 2010 and for the six most landed shark species from 2000 to 2010. Slope: parameter estimate for year; SE: standard error of predicted estimate; n.s.: not significant p-value (>=0.05); RSE: model residual standard error; upper and lower limits of the 95% confidence interval. Mean annual change in landings was calculated as = [(e ^slope^ - 1) × 100]. The upper and lower limits of change in landings was calculated as = [(e ^slope±1.96SE^ -1) × 100].

		Year Predictor					95% CI of slope		Change in landings		
Fishery	Year	Slope	SE	t-ratio	P-val	RSE	2.5%	97.5%	Mean	2.5%	97.5%
All sharks	1950–1959	0.136	0.037	3.679	0.006	0.123	0.0552	0.2198	14.6	6.6	23.2
	1960–1969	0.142	0.039	3.638	0.007	0.120	0.0427	0.2364	15.3	6.8	24.5
	1970–1979	-0.022	0.029	-0.779	n.s.	0.085	-0.1148	0.0205	0		
	1980–1989	-0.024	0.042	-0.587	n.s.	0.125	-0.1097	0.0338	0		
	1990–1999	0.030	0.039	0.765	n.s.	0.125	-0.0436	0.1189	0		
	2000–2010	-0.035	0.011	-3.281	0.010	0.039	-0.0515	-0.0039	-3.45	-5.4	-1.4
*A. vulpinus*	2000–2010	-0.013	0.055	-0.238	n.s.	0.169	-0.1258	0.1041	0		
*I. oxyrinchus*	2000–2010	-0.032	0.012	-2.774	0.022	0.033	-0.0621	-0.0142	-3.16	-5.3	-0.9
*M. whitneyi*	2000–2010	-0.026	0.033	-0.798	n.s.	0.099	-0.0686	0.0577	0		
*P. glauca*	2000–2010	-0.048	0.026	-1.847	n.s.	0.072	-0.1109	0.0371	0		
*S. zygaena*	2000–2010	0.069	0.029	2.359	0.043	0.086	-0.0075	0.1516	7.14	1.2	13.4
*S. californica*	2000–2010	-0.209	0.052	-4.048	0.003	0.158	-0.3285	-0.1407	-18.88	-26.7	-10.2

**Figure 3.**
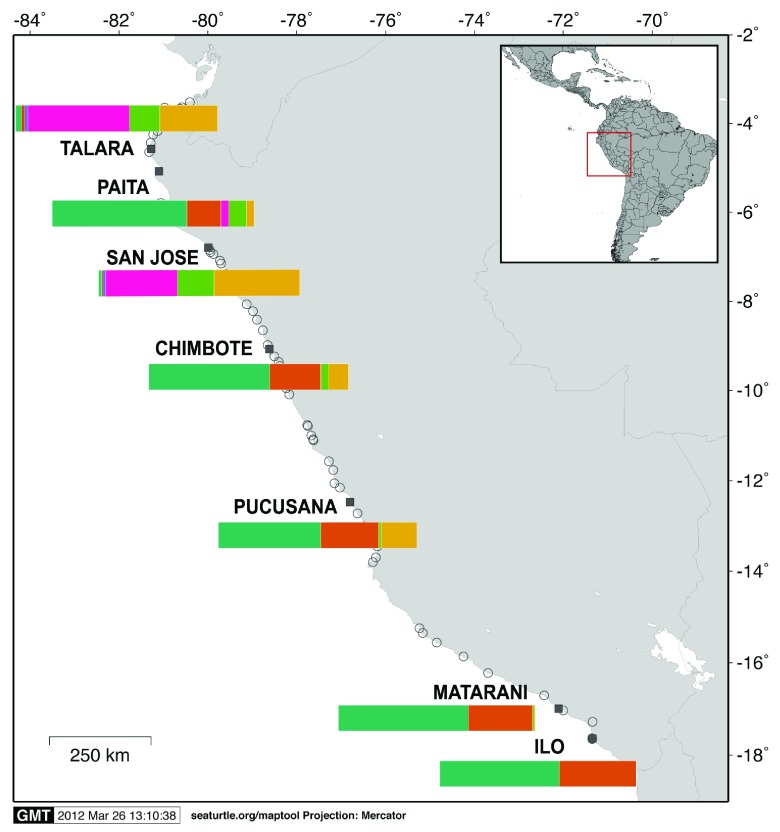
Geographic distribution of shark landing points in Peru.

Shark landings were not homogeneously distributed along the coast; we observed a tendency to land certain species at specific points ( Figure 3). The analysis was limited to the main ports in the north (i.e. Talara, Paita, San Jose), central (i.e. Chimbote and Pucusana) and south (i.e. Matarani and Ilo) for which information at species level was available from 1996 to 2010. The data suggests that the port of Ilo is the principal landing point for sharks (by biomass). An approximate of 9910 t of sharks was landed, accounting for 32% of the total biomass landed in these seven ports. The second most important port was Pucusana (21%), followed by Paita (20%), San Jose (14%), and Chimbote (9%). Talara and Matarani, together, accounted for less than 4% of the total landings. All ports landed the six species, but landings were biased towards certain shark species: blue sharks were mostly caught in southern (45%) and central (38% of total blue shark landings) landing points; shortfin mako were mostly caught in south (54%) and central (40%); smooth hammerheads were mostly caught in central (43%) and north (57%); and smooth-hound sharks, common thresher and angel sharks were mostly caught in the north with 99%, 83% and 95% landings, respectively.

Landing points reporting shark landings (open circles), including the seven points with highest landings (black squares) along the coastline of Peru with the landing composition by the six most important shark species 1996 to 2010. The horizontal bar represents total landings by species in percent. Dark green: *Prionace glauca*, orange: *Isurus oxyrinchus*, purple: *Squatina californica*, fucsia: *Mustelus whitneyi*, light green: *Alopias vulpinus*, and yellow: *Sphyrna zygaena*.

From 1996 to 2000, longline accounted for the highest percentage of landed sharks by biomass (60%), followed by gillnets (32%), purse seine (6%), and beach seine and others that together accounted for the remaining 8%. In general, the gillnet shark fishery catches the highest diversity of sharks (up to 20 species) including both pelagic and benthic species. A marked difference in the composition of the most landed shark species by each fishing method was observed: vessels used longline to capture blue shark (79% of its total landing), shortfin mako (94%) and common thresher shark (39%). In contrast, gillnets were used to capture smooth hammerhead (83%), smooth-hound shark (85%), common thresher shark (58%), and angel shark (86%).

The average CPUE (2002–2007) for the blue shark, shortfin mako and common thresher shark caught with longline were: 1.18 (SD±0.57), 0.69 (SD±0.07) and 0.07 (SD±0.04) kg of sharks, respectively, per 100 hooks per year. Furthermore, for the smooth hammerhead, smooth-hound shark, common thresher shark, and angel shark caught with gillnets were: 0.37 (SD±0.38), 0.11 (SD±0.04), 0.08 (SD±0.05) and 0.006 (SD±0.004) kg of sharks, respectively, per 100 m per year.

### Regional perspective of the Peruvian shark fishery

Throughout the Pacific, between 1950 and 2010, Peru had the sixth highest accumulated landings of chondrichthyans (sharks, batoids and chimaeras). The first five countries were (in order): Japan, Taiwan Province, Indonesia, Mexico and Republic of Korea. Along the eastern Pacific, Peru had the second highest number of landings, after Mexico. Other important countries were the USA, Chile, Canada and Costa Rica. In the southeast Pacific, Peru reported the highest landing of chondrichthyans, which was four-fold higher compared with Chile, which had the second highest number of landings in the region.

According to FAO, between 1950 and 2010 (for the 24 countries that report its landings at shark level) Peru exhibited the highest accumulated historical landings of sharks in the Pacific basin (431,534 t) followed by New Zealand, Mexico and Indonesia. In the east Pacific, Mexico’s landings were approximately half of Peru’s landings (230,986 t). However, the annual contribution of Peru to total shark landings in the Pacific has changed ( Figure 1). It increased from ~7% in 1950 to an all-time high of 88% in 1968, decreasing to a minimum of 2.95% in 2006. In the last five years assessed (2005–2010), the mean contribution was 5.88% (± 2.15), slightly higher than the contribution of Ecuador (5.50% ± 2.96) and Costa Rica (1.56 ± 0.04). In the same period, Indonesia’s mean contribution to Pacific shark landings was 47.27% (± 4.76) and New Zealand contributed 16.14% (± 2.12) of total shark landings.

### Peruvian shark fin and meat international commerce

Six different commodity codes used by SUNAT were used to identify shark-based products (0305591000, 0305710000, 0303750000, 0303810000, 0302810000, 0305791000); half of them corresponded to shark fin while the other half identified shark meat. All of them contained reports of international commerce that, when pooled together, provided all import and export movements from 1997 to 2012 by country of origin/destination.

A significant positive trend was detected for the export and import of shark fins and meat between 1997 and 2012 (shark fin import: r=0.75, p-value<0.001, export: r=0.56, p-value=0.02; shark meat import: r=0.90, p-value<0.001, export: r=0.64, p-value=0.008, see Figure 1). From 1997 to 2012, Peru imported 268.66 t of shark fin worth 2,279,003.67 US dollars and exported 2,353.70 t of shark fin worth 101,480,171.30 US dollars ( Table 3). The main origin for shark fin supply to Peru in this period of time was Ecuador (~87%), followed by the high seas (i.e. international waters, 8%), and Spain (3%). The main destination of shark fin export was Hong Kong (87%), followed by Japan (7.2%), China (~1.3%), United States (1%); minor exports (<1%) were shipped to another 22 other countries around the globe. In the same period of time, Peru imported 16113.85 t of shark meat worth 9,817,805.73 US dollars, mainly from high seas (29%), Japan (~28%), Ecuador (22%), and Spain (9%), and exported 8,177.96 t worth 12,496,756.72 US dollars mainly to Brazil (69%), Venezuela (9%), Colombia (7%), and Spain (6%).

**Table 3.** Import and export of Peruvian shark products (meat and fins) between 1997 and 2012.

	Shark fin		Shark meat		Total	
Year	Import	Export	Import	Export	Import	Export
1997		5.32		13.50		18.82
1998	0.26	156.91		857.73	0.26	1014.64
1999	0.78	114.65		360.58	0.78	475.23
2000	0.20	177.42	16.04	152.12	16.24	329.54
2001		138.78	26.23	380.97	26.23	519.74
2002		114.85	224.61	233.98	224.61	348.83
2003	0.50	110.61	1273.78	75.72	1274.28	186.32
2004	0.63	105.99	1113.84	149.86	1114.46	255.85
2005	0.84	162.36	940.25	419.25	941.09	581.62
2006	7.76	182.25	1072.37	220.58	1080.13	402.83
2007	1.22	242.35	1118.68	581.75	1119.90	824.10
2008	28.11	136.01	1478.55	383.93	1506.66	519.94
2009	49.57	157.59	1847.88	1360.79	1897.46	1518.38
2010	77.48	205.02	1661.32	916.58	1738.81	1121.60
2011	71.10	206.31	1827.62	858.85	1898.72	1065.16
2012	30.20	137.27	3512.68	1211.77	3542.88	1349.04
**TOTAL**	**268.66**	**2353.70**	**16113.84**	**8177.95**	**16382.50**	**10531.65**

### Conservation status and legal framework

Of the 33 species that interact with fisheries in Peru, two are listed as Endangered, 11 as Vulnerable, 10 as Near-Threatened, one as Least Concern, and eight are classified as Data Deficient by the Red List of the International Union of the Conservation of Nature (IUCN, www.redlist.org, accessed in March 2012) ( Table 1). According to the Peruvian government, none of the shark species in Peruvian waters are categorized as threatened; therefore they are not legally protected and its capture, transport and exportation is authorized ( El Peruano, 2014).

The life history traits exhibited by these species indicate that 23 out of the 33 species interacting with fishery in Peru have a very low resilience, and will need a minimum of 14 years to double their population, and 10 species have low resilience, needing a minimum of 4.5–14 years to duplicate their population (FishBase, www.fishbase.org) ( Table 1). The Peruvian anchovy ( *Engraulis ringens*) - the most heavily exploited fish in world history - only needs 15 months to double its population.

The whale shark ( *Rhincodon typus*), oceanic whitetip shark ( *Carcharhinus longimanus*), porbeagle shark ( *Lamna nasus*), scalloped hammerhead ( *Sphyrna lewini*), smooth hammerhead ( *Sphyrna zygaena*) and great hammerhead ( *Sphyrna mokarran*) are listed in Appendix II of The Convention on International Trade in Endangered Species of Wild Fauna and Flora (CITES, www.cites.org, accessed in March 2013). The mako shark, the porbeagle shark and the whale shark are listed in the Convention on Migratory Species (CMS, www.cms.int, April 2012), and the United Nations Convention on the Law of the Sea includes the whale shark, members of the Family Sphyrnidae (hammerhead sharks), Isuridae (mackerel sharks), and Carcharhinidae (whaler sharks) in its list of highly migratory species. Peru has been landing whale sharks since 2006; 2,813 t of whale sharks were landed as reported by IMARPE, but isolated events of unreported landings have been observed in northern Peru (B. Alcorta pers. comm.).

The shark fishery in Peru is regulated by the Ministerial Resolution (RM) N° 209-2001-PE from the Ministry of Fisheries (now Ministry of Production, Vice-Ministry of Fisheries). The RM 209-2001-PE is the most comprehensive regulation for the shark fishery in Peru. It establishes the shark minimum length that can be landed, the maximum tolerance of captured individuals under the minimum length and the minimum mesh size for gillnets targeting sharks. The minimum length at capture applies to five species and one genus: blue shark, shortfin mako, smooth-hounds ( *Mustelus whitneyi, M. mento*), spotted hound shark ( *Triakis maculata)* and species of the genus *Carcharhinus* ( Table 1).

There are additional regulations for other fisheries (e.g. sea bass, migratory species, and tuna;) that indirectly regulate the capture of sharks by considering them accompanying fauna (i.e. bycatch). According to the RM 236-2001-PE, the Peruvian government regulates the integrated management and rational exploitation of highly migratory species. IMARPE establishes that the following shark species are categorized as highly migratory: silky shark ( *Carcharhinus falciformis*), Galapagos shark ( *Carcharhinus galapagensis*), blacktip shark ( *Carcharhinus limbatus*), oceanic whitetip shark ( *Carcharhinus longimanus*), shortfin mako ( *Isurus oxyrinchus*), blue shark ( *Prionace glauca*), smooth hammerhead shark ( *Sphyrna zygaena*), Galapagos bullhead shark ( *Heterodontus quoyi*), thresher shark ( *Alopias vulpinus*) and tope shark ( *Galeorhinus galeus*) (DE-100-034-2002-IMP/PE). According to the DS 032-2003-PRODUCE, the fishery of tunas and related species (highly migratory shark species, DE-100-034-2002-IMP/PE) should be sustainable through the implementation of measures for their management and conservation. Moreover, Peru is a member of the Inter-American Tropical Tuna Commission (abbreviated IATTC). The IATTC urges its member states to cooperate through regional fishery management organizations, in order to ensure the sustainability of shark populations and to adopt a National Plan of Action for the conservation and management of sharks. Also, the IATTC disallows the retention, landing and sale of the oceanic whitetip shark. According to the RM 236-2001-PE, the Peruvian government promote the integral development of the Patagonian toothfish ( *Dissostichus eleginoides*) fishery and its accompanying fauna (Pacific sleeper shark, *Somniosus pacificus*) which enables a sustainable fishery. Moreover, the accompanying fauna should be retained and not discarded.

## Discussion

This study represents the first analysis of Peruvian shark fishery, provides a historical perspective of its dynamics over a 61-year period and sets up a baseline of information for further research. The results are discussed within a national and regional framework in the light of shark conservation status, the current legal framework in which the shark fishery operates, and the gaps of information and regulation.

### A national and regional perspective of the shark fishery

A temporal and spatial variation in shark landings was observed. During the 61 years assessed, shark landings significantly increased by nearly 15% per year during the first two decades; but during the last 11 years it has experienced a constant annual decline of more than 3%. The increase in shark landings during the first two decades might be the results of various historical factors. Since 1940 the Peruvian population has mainly migrated to the coast: 28.3% of the Peruvian population lived in the coast by 1940; while 46.1% of the population lived near the coast by 1972. So, the economically active population of the fishery sector have grown from 1435 in 1940 to 66062 in 1994 ( OIM, 2015). This coastal migration might have promoted an increase in the consumption and demand of seafood, triggering the fishery. The Peruvian fishery industrialization begun in 1950 which was developed to capture Peruvian anchovy. This might have motivated artisanal fishermen to expand the size, capacity and power of its vessels. This way, the average length of gillnets have change dramatically from the years 1970 to the period between 2002–2007: 72–81 m to 1900 m ( Alfaro-Shigueto *et al.*, 2010; Castillo, 1970). Peruvian vessels related to shark fishery have doubled from 2061 in 1996 to 4013 in 2002 ( IMARPE, 2003). The first national census of small-scale fishery reported that in 2012 the number of fishermen that target sharks were 1522. Moreover, between 1995 and 2010, the number of vessels using longline has increased by 357%; while the total length of gillnets in Peru was estimated at >100,000 km of net per year: 14 times the length used by the Taiwanese high seas driftnet fleet in the Pacific before it was banned ( Alfaro-Shigueto *et al.*, 2010). It is noteworthy that the Peruvian fleet using longline and gillnet has increased but the landings have not. This decoupling between fleet size and shark landings might be caused by the following factors or combination of factors: illegal, unreported and unregulated (IUU) fishing in Peru, oceanographic and biological conditions (e.g. El Nino Southern Oscillation, prey availability), changes in fishing target and the decline of the populations of targeted species.

The extent of IUU fishing in Peru has been determined as critical in Peruvian waters and suggested to be around 30% of total biomass landed ( Pramod *et al.*, 2008), but has not been explicitly assessed for sharks. The incidental capture (or bycatch) of sharks occurs but it is unreported. A bycatch rate of 0.99 sharks every 1000 hooks was estimated from the mahi-mahi longline fishery in four ports in Peru between 2004 and 2006, while at the port of Ilo, between 2005 and 2006, shark bycatch represented 1% of the total landings ( Gilman *et al.*, 2008). The hake and shrimp fisheries have high shark interactions and discard rates ( Kelleher, 2005; Pitcher *et al.*, 2006). In Peru, six species of sharks are incidentally captured and discarded at sea ( *Mustelus whitneyi, Mustelus* sp. *, Notorynchus cepedianus*, *Echinorhinus cookie*, *Galeorhinus galeus*, *Somniosus pacificus, Squatina* sp.; Bustamante, 1997; Cespedes, 2013). Moreover, small scale trawlers, the numbers of which are unknown but likely considerable, illegally target hake in northern Peru. To the best of our knowledge, no more data on shark bycatch is available; however, reports from fishing gears, specifically trawlers and purse seiners, could be considered as preliminary information since these fisheries do not target sharks: 1130 t of sharks were caught using purse seine (96%) and trawlers (4%) (data from IMARPE, between 1996 and 2000).

We were able to find values of fishing effort; but these were rather scarce. According to Elliot *et al.* (1995); Elliot *et al.* (1996); Elliot *et al.* (1997a); Elliot *et al.* (1997b); the CPUE in the longline shark fishery (number of individuals) in northern and central (120–250 miles off shore) Peru were: 7, 6.6, 4.5, 2.5 sharks per 100 hooks for the years 1995, 1996 and 1997a and 1997b, respectively. The CPUE (number of individuals/biomass per 100 hooks) by species were: the blue shark (5, 4.7, 2 and 1.8 individuals/123.4, 40.4, 131.6, 58.2 kg), shortfin mako shark (1.8 and 0.4 individuals/61.6 and 7.6 kg), smooth hammerhead shark (1.06 and 0.16 individuals/8.9 and 17.7 kg), thresher shark (0.13 and 0.02 individuals/8.5 and 1 kg) and copper shark (0.04, 0.4 and 1.1 individuals/48.3, 15.1 and 1.1 kg) for the years 1995, 1996 and 1997a and 1997b, respectively. Another study determine the CPUE for the Peruvian small-scale longline fishery in southern Peru (Ilo). The CPUE mean and standard deviation was 33.6 (SD± 10.9) sharks per 1000 (for the shark season) and 1.9 ± 3.1 sharks per 1000 hooks (for the dolphinfish season). Of these, 70.6% were blue sharks, 28.4% shortfin mako sharks, and 1% were other species (including thresher, hammerhead, porbeagle, and other Carcharhinidae species) ( Doherty *et al.* 2014). If we compare the values of Elliot *et al.* (1995); Elliot *et al.* (1996); Elliot *et al.* (1997a); Elliot *et al.* (1997b), with the values obtained in this study (for the species caught in longline: blue shark, shortfin mako shark, and thresher shark), the CPUE has declined likely suggesting a reduction in Peruvian shark population. Nevertheless, our estimates are an extrapolation that uses Peruvian shark total landings which might be underestimated or incorrectly assumed. Further studies should aim to calculate a more accurate and reliable CPUE.

The El Nino Southern Oscillation (ENSO) could also be influencing shark landings. A correlation between species range expansions and contractions and ENSO has been observed and likely to be caused by habitat alterations and changes in food availability (reviewed in Fiedler, 2002). Indeed, prey availability ( Vas, 1990) and sea surface temperature ( Nakano & Seki, 2003; Walsh & Kleiber, 2001) have been shown to correlate with blue sharks catches. Sharks have been reported in greater abundance as well as moving southward and closer to the coast in the southeast and northeast Pacific during ENSO events (reviewed by Alvial, 1987; Sielfeld *et al.*, 2010). Similarly, the jumbo squid ( *Dosidiscus gigas*) a species preyed by top-predators, including sharks ( Lopez *et al.*, 2010; Vetter *et al.*, 2008), exhibited changes in its biomass and distribution range as a response to the environmental changes observed during an ENSO ( Field *et al.*, 2007; Zeidberg & Robison, 2007). In this study, a weak but significant correlation between the ENSO and the biomass of four species of sharks landed along the coast of Peru was detected.

Peru reports an approximate 11% of the total diversity of sharks, exhibiting high values of shark species richness and functional richness ( Lucifora *et al.*, 2011). According to this study, more than half of the sharks reported in Peru are considered of commercial importance; however, this could be an underestimate. Some commercial species are grouped with a single common name (i.e. “toyo”) that can represent many species in at least two genera (e.g. *Mustelus spp.*, *Triakis spp.*) and that include species considered as Endangered (i.e. *T. acutipinna*, IUCN Red List). For other species, such as thresher sharks ( *Alopias* sp.), species identification based on morphology can be difficult to assess when only parts of individuals are landed, which usually occurs. Here, molecular analyses using genetic barcodes (reviewed in Bucklin *et al.*, 2011) or a barcoding approach ( Naylor *et al.*, 2012) stand as a powerful tool to identify species landed. Indeed, a study using this approach suggests a misidentification of the species actually landed ( Velez-Zuazo *et al.*, 2015).

Shark landings, at the species level, were not equally distributed by port, along the coast. This might be due to the presence of two main marine currents, the Peruvian Current (or Humboldt Current) and the Equatorial Current, and the regions identified under their influence: temperate cold upwelling region in the south, tropical warm region in the south, and an intermediate area where the two currents converge in the north of Peru ( Spalding *et al.*, 2007). Shark landings, particularly species occupying coastal habitats, and mostly caught with gillnets, might be influenced by local oceanographic characteristics and species behaviour (e.g. Espinoza *et al.*, 2011). Pelagic species (e.g. blue shark), on the other hand, might be influenced by other factors. Pellon & Cardenas, 1993 reported that in the exploratory fishery of tuna using longline, high concentrations of blue shark, shortfin mako and common thresher were found in the north off Peru. Another factor influencing landings at ports is that the use of fishing gear is skewed by zone: the central and south of Peru uses mainly longline; while gillnets are the dominant fishing gear in northern Peru. Further research is needed to understand fishing gear selectivity on shark species capture ( Pope *et al.*, 1975).

The Peruvian fishery of elasmobranchs is the third largest in the Americas ( Bonfil, 1994) and Peru is among the top 26 shark fishing countries in the world ( Fischer *et al.*, 2012). This analysis suggests that Peru has landed more sharks than any other country in the entire Pacific. It is worth noting that neither Costa Rica nor Ecuador, important countries in the eastern Pacific that export shark fins to the Asian market, were at the top of the list. In both countries, until recently, shark finning was legal and common practice, likely contributing to an underestimation of the biomass of sharks captured but discarded at sea ( Clarke *et al.*, 2006). In Ecuador, underestimation of shark landings were highlighted by Jacquet *et al.*, 2008 and their study provided new estimates from 1979 to 2004. This was compared with Peruvian estimates and similar landings were found for both countries during that period, with Ecuador having a slightly higher overall landing (178,569 t in Ecuador and 175,571 t in Peru).

Although foreign fishing vessels might have played an important role in the Peruvian shark fishery, we could only find two articles regarding the Japanese explorative fishery in Peruvian waters ( Pellon & Cardenas, 1993; Torres & Gonzalez, 1993). These reports state that since 1970 Japanese longliners have operated sporadically in Peruvian waters. The exploratory area was between 03°25´ and 17°30´ south latitude and in the west until 200 nautical miles. Sharks presented high concentrations at the west of Isla de Lobos de Tierra and at 180 miles off shore Caleta La Cruz. The following species of sharks were captured: blue shark, shortfin mako, smooth hammerhead and thresher shark. Even though these are the only papers available, many fishermen state that during the 60´ and 70´ foreign fishing vessels from Poland, Russia and Japan caught sharks in the Peruvian coast.

Regional initiatives for management and conservation followed after the 1999 FAO International Plan of Action for the Conservation and Management of Sharks (IPOA-sharks). The Regional Action Plan for the Protection and Management of the elasmobranchs of the Southeast Pacific Ocean (PAR-Tiburon) was approved in 2010 by the “Comision Permanente del Pacifico Sudeste” ( CPPS, 2010) attended by Colombia, Ecuador, Peru, and Chile. At the country level, Peru has adopted the regional plan and just approved its national plan of action (NPOA, Decreto Supremo No 002-2014-PRODUCE) for the conservation and management of elasmobranchs. National plan of action (NPOA) for the conservation and management of elasmobranchs.

### Steady growth of shark international commerce

Both shark fin and meat imports and exports exhibited a positive and significant trend during the time evaluated (1997 to 2012). There were differences, however, in the volumes. While import is much lower than export in shark fin, shark meat import is higher that export. Peru exported almost ten times the quantity of shark fins it imported (export=2,353.7 t versus import=268.66 t) and almost double the amount of shark meat (16,113.84 t) was imported compared to the amount exported (8,177.95 t). This suggests that shark fins are a commodity that Peru mostly exports while there is a local demand for shark meat. Indeed, a recent report indicates that Peru is among the top 20 importers of shark meat and is the most important in the southeast Pacific ( Mundy-Taylor & Crook, 2013). There is no doubt, however, of the profit made exporting shark fins. During the period of time analyzed, Peru exported shark fins for a FOB value of more than 101 million US dollars. During the same period, the FOB value of shark meat was only around 12.5 million US dollars, even though its export volume was four times higher than shark fins.

A recent study addressing the global shark business estimated that Peru was among the twelve major countries in the world supplying shark fins to the market of Hong Kong between 2002 and 2008 ( Cheung & Chang, 2011). CITES determined that a total of 2,768 t of sharks and an average of 146 t of shark fins per year were exported from Peru between 2003 and 2008 ( CITES, 2011). The estimates of shark fins exported in our current study are very similar although slightly higher. During that same period of time, Peru exported 156 t of shark fins per year.

The dynamics of shark fin import indicates that Ecuador is the major country of origin of fins entering Peru, starting in 2008. This is very likely explained by the change in shark fin export regulations in Ecuador ( Jacquet *et al.*, 2008). In 2004, a decree from the Ecuadorian government banned all shark fin exports, but in 2008 it was overturned. Before 2008, the average import was 1.52 t originating from several different countries with no obvious dominance over these years. In 2008, however, shark fin imports increased by almost 30-fold. Between 2008 and 2012, Peru imported an average of 51 t of shark fins, almost exclusively from Ecuador. The second most significant source of shark fins was from the high seas, with a total of 22.8 t of fins imported. Since Peru stills lacks regulation for shark finning in general, there is no control over the origin of the fins obtained from vessels fishing in the high seas and how the fins were obtained, although shark finning is banned in the eastern Pacific ocean ( IATTC, 2005). While Ecuador is the main source of shark fin imports, Hong Kong is the main destination for shark fin export. Nearly 87% of shark fins exported between 1997 and 2012 went to Hong Kong. Five other countries in Asia made up a further 10% (i.e. China, Japan, Singapore, Republic of Korea, and Vietnam).

The import and export of shark meat has experienced steady growth. In 1997 Peru imported 16 t and in 2012 imported 5959 t. An important source is the high seas, which provided nearly 36% of all shark meat imported by Peru. Other important sources were Japan, Ecuador and Spain. While Asia was the most important destination area for shark fins, it was Latin America that was the most important area for shark meat exports. Nine countries, headed by Brazil (70%), accounted for 90% of all exports shipped from Peru between 1997 and 2012.

### Conservation status and legal framework of sharks interacting with fisheries

Peruvian shark fishery is regulated; however, this regulation has three fundamental flaws. First, only 13 of the 33 commercially fished species and merely four of the 13 species that are threatened are included, leaving the fishery of species such as the smooth hammerhead unregulated. Neonates and juvenile hammerheads (i.e. those under 90 cm in length) are targeted heavily in the north of Peru ( Gonzalez-Pestana, 2014). Between 1991 and 2000, 97% of smooth hammerheads landed in the port of San Jose had not reached sexual maturity ( Castañeda, 2001). The listing of *S. zygaena* in CITES Appendix II in March 2013 represents a new challenge and task for Peru for regulating and controlling the international trade of this species, which is currently fished without any kind of regulation. To reinforce the scale of this fishery, between 2002 and 2011, Peru landed a higher biomass of smooth hammerheads than the sum of all other countries reporting landings for this species (i.e. Spain, Ecuador, Portugal, and New Zealand; Mundy-Taylor & Crook, 2013). Second, it sets the minimum capture length only for a handful of species, which does not include three out of the six most landed sharks (i.e. smooth hammerhead, common thresher, and angel sharks) and groups other species by genus, setting the minimum size for the whole group. This is the case of the genus *Carcharhinus*, where the minimum capture length is 150 cm; however, of the eight species of *Carcharhinus* reported interacting with fisheries in Peru, two species mature at smaller sizes, therefore, catches are only allowed for mature individuals, which potentially has a negative impact on their populations. The regulation of the minimum capture length leaves open and unregulated the fishery on adults, which is detrimental for the population. The protection and management of a shark breeding area does not guarantee that the population is healthy in the absence of a lack of management for other size classes ( Kinney & Simpfendorfer, 2009). Demographic models and experiences in the shark fishery management indicate that focusing the management on adults would be more beneficial for the total population ( Brewster-Geisz & Miller, 2000; Gallucci *et al.*, 2006; Musick, 1999). For example, over 30 years the management of tope shark fishery in South Australia was oriented toward protecting their breeding areas. However, the population declined until the fishery collapsed. This was because the adults were fished unsustainably during those 30 years ( Kinney & Simpfendorfer, 2009). Taking this as an experience, the *Mustelus antarcticus* fishery in southeast Australia focused their effort on a single class, youth, resulting in a sustainable fishery ( Prince, 1992). This way, the minimum capture length regulation should be re-evaluated. Third, the regulation is not implemented and enforced; foremost many of the fishermen are unaware of it. The minimum capture length for the mako shark is set at 170 cm but IMARPE reports that 60.1% (year 2009) and 88.5% (2010) of mako landings in the port of Pucusana were under minimum legal size ( Anuario CientíficoTecnológico Vol. 5–10, 2005–2010). Of the 41 fishermen interviewed (in the landing points of Paita, Salaverry and Ilo), 93% do not discard any shark once it is hooked, only 34% reported that they discard sharks below 40–60 cm, and only 4.8% were aware of the regulations (Mangel *et al.*, 2007). In Peru there are two marine protected areas: Paracas and the Sistem of Islands, Islets and Capes. Sustainable fishery is allowed in these areas, but not frequently enforced. Currently, the presence and distribution of sharks in these areas is unreported.

Peru has no regulation against shark finning (i.e. the practice of removing and retaining the fins of live sharks and returning the shark back to the sea where it eventually dies), but finning at sea seems not to occur in Peru. Gilman *et al.* (2008) and field observations agree that sharks are commonly landed with fins attached and later removed and sold at the port. Shark fins in Peru have a price by kilogram that is higher compared to the rest of the carcass due to their international demand to prepare shark fin soup. In 2011, the minimum price of shark fin in the local market was 64.2 US$ per kilogram while the maximum price was 98.58 US$. Shark fins, as estimated in this study, are one of most important products exported. Even though finning occurs rarely in Peru; we recommend to discourage potential attempts to make it a common practice.

### Gaps of information and recommendations

Research is urgently needed to improve the conservation of sharks and should include taxonomy (production of identification guides), life history (e.g. reproductive biology), spatial ecology (e.g. movement and migration), effect of climate variability (e.g. ENSO), ecosystem role (e.g. diet and trophic structure), fishing (e.g. catch and effort), population status (e.g. stock assessment), commerce (e.g. commercial routes of shark fins), and human values, attitudes, beliefs and behaviors toward sharks ( Simpfendorfer *et al.*, 2011). This information is essential to design and implement shark management (e.g. fishery and marine protected areas). For example, combining fishery with biological and ecological information is crucial to estimate the number of individuals that could be safely removed without affecting the integrity and functionality of populations ( Botsford *et al.*, 1997; Dankel *et al.*, 2008; Köster *et al.*, 2003). Understanding the trophic ecology of marine environments where sharks interact is critical for implementing ecosystem based fishery management ( FAO, 1996) that is being applied by IMARPE. Also, it is equally important to understand the values, attitudes, beliefs and behaviors of the people, industries and communities that depend on sharks ( Simpfendorfer *et al.*, 2011) because managing resources is also about the people who exploit it ( Hilborn, 2007).

With so many species interacting with fishery at different spatial and temporal scales Peru must prioritize its conservation management on the most fished and imperiled species. First, management should be applied to the six most fished species in Peru at the most important landing points. Legal regulation is a critical element in achieving effective conservation and management of sharks ( Techera & Klein, 2011). Thus, shark finning must be banned and NPOA must be implemented in the short term. One of the major challenges for the conservation of sharks is that the most caught species in Peru are also highly migratory (i.e. blue shark, mako shark, smooth hammerhead and common thresher). So, they are constantly crossing nation borders; if one country within the migratory range has weaker laws, it may undermine conservation and management efforts in another ( Techera & Klein, 2011). As such, regional actions are necessary.

## Conclusions

The Peruvian fishery interacts with 33 shark species of which the six most frequently landed are the blue shark, shortfin mako shark, smooth hammerhead, smooth-hound shark, common thresher, and the angel shark. The highest landings occur at Talara, Paita, San Jose, Chimbote, Pucusana, Matarani and Ilo; and the fishery gear most used are longline and gillnets. Peru is one of the most important countries in the whole Pacific Ocean with regards to shark fishery and the data suggests that, historically, it has landed more sharks than any other country in the Pacific. The international commerce of Peruvian shark fin and meat has increased between 1997 and 2012 with higher profits reported for exporting shark fins, mainly to Hong Kong. Peru also imports shark fin, mostly from Ecuador, although imports from the high seas represent an important source of fins and meat and this needs attention to reduce the entrance of IUU fishery products. The Peruvian shark fishery is under-monitored and under-regulated; even though 41% of shark species landed are threatened and seven species that interact in Peruvian fishery are included in CITES and CMS. The most comprehensive shark regulation in Peru, Ministerial Resolution N° 209-2001-PE, has fundamental flaws that need to be addressed. Great gaps in information exist that hampers its management including in basic taxonomy, life history and spatial ecology, among others, but this could be addressed by promoting research, education and awareness and involving stakeholders at all levels. Peru has made the first steps towards the recovery and sustainable conservation of sharks by approving the NPOA, but works remains to be done.

## Data availability

The data referenced by this article are under copyright with the following copyright statement: Copyright: © 2016 Gonzalez-Pestana A et al.

Data associated with the article are available under the terms of the Creative Commons Zero "No rights reserved" data waiver (CC0 1.0 Public domain dedication). 

Access to the landing statistics collected by the Instituto del Mar del Peru is possible by presenting, in person, at the administrative office, providing a filled form (downloadable from here: http://www.imarpe.pe/imarpe/index.php?id_seccion=I0116010601000000000000) and corresponding fee, following the instructions described here: http://www.imarpe.pe/imarpe/archivos/informes/imarpe/tup_mod_rd_de_125_2010.xls, also accessible here: http://www.imarpe.pe/imarpe/index.php?id_seccion=I0116010601000000000000 All documents are in Spanish.

Access to landing statistics published by FAO are available in their webpage ( www.fao.org) using the software FishStatJ v.2.0.0 ( http://www.fao.org/fishery/statistics/software/en).
