# Supplementary material for: Shark fisheries in the Southeast Pacific: A 61-year analysis from Peru
Source: F1000Res. 2016 Apr 12;3:164. Originally published 2014 Jul 21. [Version 2] doi: 10.12688/f1000research.4412.2 (PMC5017296; doi:10.12688/f1000research.4412.2)
Supplement: Peer review history

# Peer review: Referee response for version 2

**DOI:** 10.5256/f1000research.8877.r16077

**Published:** 2016-09-06

**Authors:** Shelley C. Clarke

**Affiliations:** 1 ABNJ Tuna Project (Common Oceans), Western and Central Pacific Fisheries Commission, Pohnpei, Micronesia, Federated States of

I have read the revised paper and appreciate the efforts of the authors to make significant improvements.  I also applaud the journal for making the peer review process transparent to all. This paper is a key reference for anyone interested in historical and current shark exploitation in Peru or in the Eastern Pacific region as a whole. The authors’ compilation of unpublished information in Spanish is an important contribution to the shark conservation and management community. However, historical data can often be difficult to interpret due to missing information or changing data quality over time. I feel that the authors have done what they can to formulate interesting hypotheses based on the historical data available but there remain some problems with the technical details of the analyses or the data itself which caution against accepting some of the conclusions presented. Readers should keep this in mind when evaluating the arguments put forth, and particularly when citing this paper.  Specific examples include:

  

- **Catch data vs catch rate data**. I originally suggested converting catch to catch rate to provide a time series (trend) of abundance. It appears the authors were unable to convert annual catch to annual catch rate because annual effort statistics (or their proxies) were not available. The point estimates of catch rate now provided may be useful in some sense but in a rather limited way compared to what I had been suggesting. 
-  
**Analysis of catch trends in 10-year intervals**. I understand what the authors were attempting to achieve here, but 10-year intervals are arbitrary, and potentially misleading if important changes in the fishery (or the way data are recorded) occur in the midst of these intervals. I consider it would have been better to simply describe the trends in a qualitative fashion. 
- **Potential bias due to reporting practices**. I am not sure whether in Peru as in most countries there have been shifts toward more species-specific reporting over time. This can be seen in “fish” being increasingly reported as for example “chondrichthyan fish”, “chondrichthyan fish” being reported as for example “sharks” (or “rays”), and “sharks” being reported to species. It is also possible that the coverage of fishery statistics improved over time. It would have been good to document how these factors apply to Peru and whether change occurred across the whole country at the same time. The likelihood that these types of changes did not occur synchronously across countries makes the international comparisons drawn in the paper problematic and potentially unreliable. 
- **Recommendations about management**. I have no doubt that shark management could be improved in Peru and appreciate the authors raising a number of suggestions for what should be done.  However, I am still not clear on exactly what the authors’ top priorities would be and why, and I would have liked to have seen a more insightful analysis. For example, it's clear the authors think the minimum size limit is not sufficient but under the situation they describe what they would like to see instead is not well-explained.  Also with the high demand for shark meat in Peru, why do the authors feel that “discouraging potential attempts to make [shark finning] a common practice” is necessary? (Note: the authors define shark finning as “the practice of removing and retaining the fins of live sharks and returning the shark back to the sea where it eventually dies” but finning can occur even if the shark is dead and finning by definition occurs at sea (see authors’ following text)).

I have read this submission. I believe that I have an appropriate level of expertise to confirm that it is of an acceptable scientific standard, however I have significant reservations, as outlined above.

# Peer review: Referee response for version 2

**DOI:** 10.5256/f1000research.8877.r16075

**Published:** 2016-09-05

**Authors:** Juan Carlos Pérez-Jiménez

**Affiliations:** 1 Department of Sustainability Science, El Colegio de la Frontera Sur, Lerma, Mexico

This is a comprehensive study on the shark fishery from Peru. I read the comments of the previous reviewers and I consider that authors take into account most of their recommendations; however, the manuscript requires some clarifications.

- Title and abstract are appropriate for the content of the article.
- Methods are partially appropriate. Analysis of the catch trends are appropriate due to the lack of effort records. However, a problem is the estimating CPUE; it is not clear how the authors extrapolated from the fishing effort of the small-scale fishery to the small-scale shark fishery.
- Also, I agree with one of the previous reviewers who “interpret that the study describe records of shark landings from any fishery in the Peruvian waters”. The comment of the authors to this interpretation was as follows: “the reports of the Peruvian small-scale shark fishery is composed of target sharks species but in some cases sharks are not the main target”.Therefore, I have two questions: where and when sharks are not the main target? This occurred in a higher or lower percent of the landings of the small-scale shark fishery? This information is important to estimate the CPUE; it was considered for the extrapolation? If the extrapolation is difficult, I recommend to eliminate the CPUE estimations; but indicate where and when sharks are not the main target of the fishing fleet (if possible).
- I consider that the small-scale fishery cannot be considered as a small-scale shark fishery, because not always are the sharks the target species; or can be considered as a “seasonal” shark fishery. It seems that small-scale fleet operates according to the seasonal abundance of several fishery resources, including sharks.

I have read this submission. I believe that I have an appropriate level of expertise to confirm that it is of an acceptable scientific standard.

# Peer review: Referee response for version 1

**DOI:** 10.5256/f1000research.4722.r6465

**Published:** 2014-12-11

**Authors:** Alastair Harry

**Affiliations:** 1 Centre for Sustainable Tropical Fisheries and Aquaculture, James Cook University, Townsville, Australia

This manuscript synthesises, reviews and analyses information on shark landings, fishery characteristics, fisheries management and trade in Peru. Production by shark fisheries in Peru has evidently been and continues to be substantial, and there is clearly a need for work such as this that brings together information and identifies knowledge gaps. The manuscript is interesting, well-researched and includes broad-scale and species-specific data from a range of sources including unpublished and government reports. It is a useful addition to the literature provides a good starting point for further research.

Although generally well-organised and structured, in parts the manuscript itself reads more like a report. More critical interpretation of the data is required and careful consideration should be given to the information that is presented. Quantitative analyses are carried out to identify statistically significant trends in the landings data and these seem fairly arbitrary and uninformative. A more useful approach would be to spend more time researching what the historical factors causing these trends are (e.g. economic, market, policy, reporting) and explaining them qualitatively. The correlation of catch with ENSO interesting but quite exploratory and I didn’t really see how it fits in with the aims of the manuscript.

A major limitation of the manuscript is a lack of any measure of fishing effort. In the discussion there is some mention of the total lengths of gillnets used and number of fishing vessels suggesting some information may be available. Even gross statistics such as these could be instructive for understanding trends in the landings data and may be worth presenting.

Also, perhaps I didn’t read carefully enough, but one thing that still wasn’t clear to me after reading is exactly what the ‘Peruvian shark fishery’ is in the context of the manuscript. Are pelagic species such as blue shark and mako taken as bycatch in pelagic fisheries for tuna/swordfish etc, or are they specifically targeted? Likewise, are *Mustelus *and *Squatina *species bycatch in groundfish fisheries, or are they targeted? My interpretation was that the manuscript was describing all records of shark landings from any ‘fishery’ in Peruvian waters, but maybe I have misinterpreted this completely.

I feel there could be more detailed discussion of the legal framework. In addition to the ‘RM’ fisheries legislation, are there any other higher-level pieces of environmental legislation that provide any legal protection for sharks? References are made to a spatial closure for angel sharks. Is this in Peru, and are there any other marine parks in Peruvian waters? Are there other management measures (e.g. limited entry) that may have direct/indirect effects on shark landings?

I have read this submission. I believe that I have an appropriate level of expertise to confirm that it is of an acceptable scientific standard, however I have significant reservations, as outlined above.

# Response

**Published:** 2016-03-03

**Authors:** Ximena Velez-Zuazo

**Affiliations:** ecOceanica and University of Puerto Rico, Peru

**Although generally well-organized and structured, in parts the manuscript itself reads more like a report. More critical interpretation of the data is required and careful consideration should be given to the information that is presented. Quantitative analyses are carried out to identify statistically significant trends in the landings data and these seem fairly arbitrary and uninformative. A more useful approach would be to spend more time researching what the historical factors causing these trends are (e.g. economic, market, policy, reporting) and explaining them qualitatively.**

In the Discussion section we included:

*“The increase in shark landings between 1950 and 1975 might have several historical factors. Since 1940 the Peruvian population has mainly migrated to the coast.  By 1940, 28.3% of the Peruvian population lived in the coast; while by 1972, 46.1% of the population lived near the coast (OIM, 2015). Therefore, the economically active population of the fishery sector have grown from 1435 in 1940 to 66062 in 1994 (INEI). This coastal migration might have motivated an increase in the consumption and demand of sea food which might have triggered the fishery. Another factor might have been the industrialization of the Peruvian fishery which begun in 1950. This was driven by the fishery of anchovy (Engraulis ringens). Therefore, these factors might have motivated the expansion in size, capacity and power of Peruvian small-scale shark fishery: 32.6m ^3^ GRT, up to 15m, motorized and voyage trips for up to 2 weeks.”*

**The correlation of catch with ENSO interesting but quite exploratory and I didn’t really see how it fits in with the aims of the manuscript.**

We explain that the ENSO might have influenced the fishery trends.

**A major limitation of the manuscript is a lack of any measure of fishing effort. In the discussion there is some mention of the total lengths of gillnets used and number of fishing vessels suggesting some information may be available. Even gross statistics such as these could be instructive for understanding trends in the landings data and may be worth presenting.**

In the Methods section we included:

*“We estimated the average catch per unit effort (CPUE) for the most landed shark species between 2002 and 2007. According to Alfaro-Shigueto et al. (2010), the fishing effort of the small scale fishery that uses gillnets is 100 000 km of nets per annum and for the longline the effort is 80 million hooks set per annum. We extrapolated these numbers to the Peruvian small-scale shark fishery so we can obtain an estimated CPUE. First for each of the most landed species, we calculated the landings per year using a particular fishing gear (gillnet or hooks). Second, we calculated the CPUE (per species, year and fishing gear): shark species biomass per 100 m per year and shark species biomass per 100 hooks per year. Finally, we obtain an average CPUE for the years between 2002 and 2007."*

In the result section we included:

*“The average CPUE (2002-2007) for the blue shark, shortfin mako and common thresher shark caught with longline were: 1.18 (SD±0.57), 0.69 (SD±0.07) and 0.07 (SD±0.04) kg of sharks, respectively, per 100 hooks per year. Furthermore, for the smooth hammerhead, smooth-hound shark, common thresher shark, and angel shark caught with gillnets were: 0.37 (SD±0.38), 0.11 (SD±0.04), 0.08 (SD±0.05) and 0.006 (SD±0.004) kg of sharks, respectively, per 100 m per year.”*

In the Discussion section we included:

*“We were able to find some values of fishing effort; but there were mainly scarce. According to Elliot et al. (1995, 1996, 1997a, 1997b), the CPUE in the longline shark fishery (number of individuals) in northern and central (120-250 miles off shore) Peru were: 7, 6.6, 4.5, 2.5 sharks per 100 hooks for the years 1995, 1996 and 1997a and 1997b, respectively.  The CPUE (number of individuals/ biomass  per 100 hooks) by species were:  the blue shark (5, 4.7, 2 and 1.8 individuals/ 123.4, 40.4, 131.6, 58.2 kg), shortfin mako shark (1.8 and 0.4 individuals/ 61.6 and 7.6 kg), smooth hammerhead shark (1.06 and 0.16 individuals/ 8.9 and 17.7 kg),  thresher shark (0.13 and 0.02 individuals / 8.5 and 1 kg) and  copper shark (0.04, 0.4 and 1.1 individuals/ 48.3, 15.1 and 1.1 kg) for the years 1995, 1996 and 1997a and 1997b, respectively.*

*Another study determine the CPUE for the Peruvian small-scale longline fishery in southern Peru. The CPUE mean and standard deviation was 33.6 (SD± 10.9) sharks per 1000 (for the shark season) and 1.9 ± 3.1 sharks per 1000 hooks (for the dolphinfish season). Of these, 70.6% were blue sharks, 28.4% shortfin mako sharks, and 1% were other species (including thresher, hammerhead, porbeagle, and other Carcharhinidae species.*

*If we compared the values of Elliot et al. (1995, 1996, 1997a, 1997b) with the values obtained in this study (for the species caught in longline: blue shark, shortfin mako shark, and thresher shark), the CPUE has declined that might suggest a reduction in Peruvian shark population. Nevertheless, our estimates are an extrapolation that uses Peruvian shark total landings which might be underestimated or incorrectly assumed; therefore further studies should calculate a more accurate and reliable CPUE.”*

*If we compared these values with the values obtained on this study (for the species caught in longline: blue shark, shortfin mako shark, and thresher shark), the CPUE has declined that might suggest a reduction in Peruvian shark population. Nevertheless, more detailed and precise studies are needed in order to corroborate these estimates.” Extrapolated, over assumptions. Currently the fleet has changed.*

**Also, perhaps I didn’t read carefully enough, but one thing that still wasn’t clear to me after reading is exactly what the ‘Peruvian shark fishery’ is in the context of the manuscript. Are pelagic species such as blue shark and mako taken as bycatch in pelagic fisheries for tuna/swordfish etc, or are they specifically targeted? Likewise, are Mustelus and Squatina species bycatch in groundfish fisheries, or are they targeted? My interpretation was that the manuscript was describing all records of shark landings from any ‘fishery’ in Peruvian waters, but maybe I have misinterpreted this completely.**

In the Introduction section we specified that the “Peruvian shark fishery” is Peruvian small-scale shark fishery (PSSSF). In the Methods section we defined small-scale fishery:

*"According to the Peruvian fisheries regulations, the small-scale fisheries (SSF) are defined as containing boats with a maximum of 32.6m ^3^ Gross Registered Tonnage (GRT), up to 15m of length and operating predominantly using manual work (El Peruano, 2001).*

*The PSSSF includes not only target and bycatch species but also oceanic and coastal species. Nevertheless, most species caught by the PSSF are* *landed and commercialized*. *The reports of the Peruvian small-scale shark fishery is composed of target shark species but in some cases sharks are not the main target. For example, in Tumbes- northern Peru- the pelagic coastal fishery mainly targets Pacific harvestfish (Peprilus medius) but neonates and juvenile hammerhead sharks are frequently caught; and for the benthic coastal fishery the main target is hake (Merluccius gayi peruanus) but humpback smoothhounds (Mustelus whitneyi) are caught. Nevertheless, sharks are not discarded; they are landed, commercialized and consumed.*

*The only PSSSF that reports shark bycatch is the Peruvian fishery of Patagonian toothfish (Dissostichus eleginoides) where the Pacific sleeper shark (Somniosus pacificus) is a bycatch (Bustamante, 1997 ).”*

**I feel there could be more detailed discussion of the legal framework. In addition to the ‘RM’ fisheries legislation, are there any other higher-level pieces of environmental legislation that provide any legal protection for sharks? References are made to a spatial closure for angel sharks. Is this in Peru, and are there any other marine parks in Peruvian waters? Are there other management measures (e.g. limited entry) that may have direct/indirect effects on shark landings?**

In the results section (Conservation status and legal framework) we included:

" *According to the Peruvian government, none of the shark species in Peruvian waters are categorized as threatened; therefore they are not legally protected and its capture, transport and exportation is authorized (El Peruano, 2014). According to RM 236-2001-PE, the Peruvian government regulates the integrated management and rational exploitation of highly migratory species. IMARPE establishes that the following shark species are categorized as highly migratory: silky shark (Carcharhinus falciformis), Galapagos shark (Carcharhinus galapagensis), blacktip shark (Carcharhinus limbatus), oceanic whitetip shark (Carcharhinus longimanus), shortfin mako (Isurus oxyrinchus), blue shark (Prionace glauca), smooth hammerhead shark (Sphyrna zygaena), Galapagos bullhead shark (Heterodontus quoyi), thresher shark (Alopias vulpinus) and tope shark (Galeorhinus galeus) (DE-100-034-2002-IMP/PE). According to the DS 032-2003-PRODUCE, the fishery of tunas and related species (highly migratory shark species, DE-100-034-2002-IMP/PE) should be sustainable through the implementation of measures for their management and conservation. Moreover, Peru is a member of the Inter-American Tropical Tuna Commission (IATTC). The IATTC urges its member states to cooperate through regional fisheries management organizations, in order to ensure the sustainability of shark populations and to adopt a National Plan of Action for the conservation and management of sharks. Also, the IATTC disallows the retention, landing and sell of the oceanic whitetip shark (Carcharhinus longimanus). According to the RM 236-2001-PE, the Peruvian government promotes the integral development of the Patagonian toothfish (Dissostichus eleginoides) fishery and its accompanying fauna (Pacific sleeper shark, Somniosus pacificus) which enables a sustainable fishery. Moreover, the accompanying fauna should be retained and not discarded. In Peru there are two marine protected areas: Paracas and Sistem of Islands, Islets and Capes. Sustainable fishery is permitted in these areas; nevertheless, it is not frequently enforced. Currently, the presence and distribution of sharks in these areas is unknown."*

# Peer review: Referee response for version 1

**DOI:** 10.5256/f1000research.4722.r5526

**Published:** 2014-08-18

**Authors:** Shelley C. Clarke

**Affiliations:** 1 ABNJ Tuna Project (Common Oceans), Western and Central Pacific Fisheries Commission, Pohnpei, Micronesia, Federated States of

This paper presents a comprehensive and very useful introduction to the shark fisheries of Peru, a topic about which very little has been published until now. The authors have expended considerable effort to compile historical data and I agree with them that this paper " *sets up a baseline for future research*". However, some of the conclusions in the paper lack sufficient justification to be reliable and should be presented as hypotheses for further study rather than findings from this paper.

The main shortcoming of the datasets used in the paper is the over-reliance on landings data to represent the state of the shark stocks. Landings data are a function of fishing effort and the recording system (coverage, consistency and species-specificity). These topics are mentioned but not given the depth of discussion that they deserve. Lack of effort data is a common problem in historical fisheries datasets and the authors may have extracted all the useful information they could from available records, but the paper does provide some information on trends in vessel numbers over time. It would have been useful to provide such numbers in a table or figure, or further to make some assumptions about the number of fishing days based on trip length for certain vessel classes and the number of landings recorded. Even a rudimentary estimate of effort would have assisted in converting catch data to catch rate and thus provide a potential index of abundance. Furthermore, it seems there could have been more discussion on how the coverage and consistency of the landings data changed over the 61 years of the study period, and when species-specific recording practices were implemented for each of the main shark species examined. 

Given that only landings data were used, and as explained above biases are largely unknown, the complexity of the analysis is probably unwarranted. It is not made clear why trends were analyzed as 10-year intervals and why this is considered appropriate. Fitting a linear model to a noisy time series is not an appropriate means of determining a trend. Even if the fit is statistically significant, the slopes and correlation coefficients are often very small and this does not support a conclusion that a meaningful trend is present. It is noted that only half of the twelve species/time intervals analyzed had statistically significant slopes, though all are presented and discussed.

The findings regarding the proportion of Peru's landings of chondrichthyans in the Pacific from 1950-2010 should be checked. Using the FAO FISHSTAT database, my calculations show that Peru's highest proportion (17%, not 80% as suggested by Figure 1) occurred in 1984 and over the 60-year time period it averaged 6%.  Of course, the figures from other countries are also subject to the time-dependent biases in fishing effort and reporting systems as mentioned for Peru above. Therefore, this comparison should be heavily caveated. 

With regard to the discussion of trade, it would have been better to present annual values (perhaps in a table) rather than aggregated amounts for 1997-2012. The important point about Peru having picked up the trade in shark fins when Ecuador banned this trade in 2008 should have been illustrated in a table or figure. I wonder if the authors were able to explore how much of the Peruvian imports during this period were from fishing vessels choosing to land shark products in Peru versus from traders re-routing their consignments through Peru. What role do foreign fishing vessels play in Peruvian shark landings and has this changed over time? It would also have been interesting to hear about the trade in skate and ray wings, and whether this follows similar patterns as shark fins or is primarily for the domestic market. 

In terms of management, the authors should be clear in recommending whether a maximum size or a minimum size is a better strategy--the discussion seems to make points on both sides of this argument. Also, what is the status of implementation and enforcement in Peruvian fisheries? Finally, since the authors claim that shark finning is not practiced because sharks are utilized for their meat, it is not clear why a shark finning ban is recommended as a management priority. It seems under current circumstances in Peru that recommending catch limits might be a more effective way of controlling shark mortality within sustainable limits.

I have read this submission. I believe that I have an appropriate level of expertise to confirm that it is of an acceptable scientific standard, however I have significant reservations, as outlined above.

# Response

**Published:** 2016-03-03

**Authors:** Ximena Velez-Zuazo

**Affiliations:** ecOceanica and University of Puerto Rico, Peru

**The main shortcoming of the datasets used in the paper is the over-reliance on landings data to represent the state of the shark stocks. Landings data are a function of fishing effort and the recording system (coverage, consistency and species-specificity). These topics are mentioned but not given the depth of discussion that they deserve. Lack of effort data is a common problem in historical fisheries datasets and the authors may have extracted all the useful information they could from available records, but the paper does provide some information on trends in vessel numbers over time. It would have been useful to provide such numbers in a table or figure, or further to make some assumptions about the number of fishing days based on trip length for certain vessel classes and the number of landings recorded. Even a rudimentary estimate of effort would have assisted in converting catch data to catch rate and thus provide a potential index of abundance.**

We agree with the reviewer that estimates of fishing effort would provide a more accurate picture of the dynamics of the shark fishery in Peru. These estimates are collected by the government but are not available to the public and are very difficult to obtain. We have made, however, an effort to get a rudimentary estimate using data from published articles. We used data from Alfaro-Shigueto et al. (2010) and extrapolated it. The details and results are now included in the Methods and Results section.

In the Methods section we included:

*"We estimated the average catch per unit effort (CPUE) for the most landed shark species between 2002 and 2007. According to Alfaro-Shigueto et al. (2010), the fishing effort of the small scale fishery that uses gillnets is 100 000 km of nets per annum and for the longline the effort is 80 million hooks set per annum. We extrapolated these numbers to the Peruvian small-scale shark fishery so we can obtain an estimated CPUE. First for each of the most landed species, we calculated the landings per year using a particular fishing gear (gillnet or hooks). Second, we calculated the CPUE (per species, year and fishing gear): shark species biomass per 100 m per year and shark species biomass per 100 hooks per year. Finally, we obtain an average CPUE for the years between 2002 and 2007."*

In the result section we included:

*“The average CPUE (2002-2007) for the blue shark, shortfin mako and common thresher shark caught with longline were: 1.18 (SD±0.57), 0.69 (SD±0.07) and 0.07 (SD±0.04) kg of sharks, respectively, per 100 hooks per year. Furthermore, for the smooth hammerhead, smooth-hound shark, common thresher shark, and angel shark caught with gillnets were: 0.37 (SD±0.38), 0.11 (SD±0.04), 0.08 (SD±0.05) and 0.006 (SD±0.004) kg of sharks, respectively, per 100 m per year.”*

In the Discussion section we included:

*“We were able to find some values of fishing effort; but there were mainly scarce. According to Elliot et al. (1995, 1996, 1997a, 1997b), the CPUE in the longline shark fishery (number of individuals) in northern and central (120-250 miles off shore) Peru were: 7, 6.6, 4.5, 2.5 sharks per 100 hooks for the years 1995, 1996 and 1997a and 1997b, respectively. The CPUE (number of individuals/ biomass per 100 hooks) by species were:  the blue shark (5, 4.7, 2 and 1.8 individuals/ 123.4, 40.4, 131.6, 58.2 kg), shortfin mako shark (1.8 and 0.4 individuals/ 61.6 and 7.6 kg), smooth hammerhead shark (1.06 and 0.16 individuals/ 8.9 and 17.7 kg),  thresher shark (0.13 and 0.02 individuals / 8.5 and 1 kg) and  copper shark (0.04, 0.4 and 1.1 individuals/ 48.3, 15.1 and 1.1 kg) for the years 1995, 1996 and 1997a and 1997b, respectively.*

*Another study determine the CPUE for the Peruvian small-scale longline fishery in southern Peru. The CPUE mean and standard deviation was 33.6 (SD± 10.9) sharks per 1000 (for the shark season) and 1.9 ± 3.1 sharks per 1000 hooks (for the dolphinfish season). Of these, 70.6% were blue sharks, 28.4% shortfin mako sharks, and 1% were other species (including thresher, hammerhead, porbeagle, and other Carcharhinidae species* *(Doherty et al. 2014)* *.*

*If we compared the values of Elliot et al. (1995, 1996, 1997a, 1997b) with the values obtained in this study (for the species caught in longline: blue shark, shortfin mako shark, and thresher shark), the CPUE has declined that might suggest a reduction in Peruvian shark population. Nevertheless, our estimates are an extrapolation that uses Peruvian shark total landings which might be underestimated or incorrectly assumed; therefore further studies should calculate a more accurate and reliable CPUE."*

**Furthermore, it seems there could have been more discussion on how the coverage and consistency of the landings data changed over the 61 years of the study period, and when species-specific recording practices were implemented for each of the main shark species examined.**

We have included a short paragraph in the Discussion section (subsection: Gaps of information and recommendations) discussing how the coverage, consistency of the landing data has change and how is currently reported by the government (i.e. Instituto del Mar del Peru and Vice-Ministry of Fisheries). 

**Given that only landings data were used, and as explained above biases are largely unknown, the complexity of the analysis is probably unwarranted. It is not made clear why trends were analyzed as 10-year intervals and why this is considered appropriate. Fitting a linear model to a noisy time series is not an appropriate means of determining a trend. Even if the fit is statistically significant, the slopes and correlation coefficients are often very small and this does not support a conclusion that a meaningful trend is present. It is noted that only half of the twelve species/time intervals analyzed had statistically significant slopes, though all are presented and discussed.**

We agree with the Reviewer that the data is noisy and biases are largely unknown. However, in order to discuss the behavior of shark landings over 61 years, we needed it an analytical approach that could be applied to all the time series equally. For this, we established a 10-year interval as fixed window to analyze the trend of shark landings to reduce the noise signal as much as possible. Larger intervals did not provide any insight, but it is clear by looking at Figure 1 that landings exhibited a trend over time period of time analyzed. Having said so, we are open to suggestions of better analytical approaches considered the data we have.

The low values for the slope and correlation coefficients were observed when investigating the influence of El Nino Southern Oscillation on shark landings. For this, we used monthly landings and compared it to Multivariate ENSO Index (MEI).

**The findings regarding the proportion of Peru's landings of chondrichthyans in the Pacific from 1950-2010 should be checked. Using the FAO FISHSTAT database, my calculations show that Peru's highest proportion (17%, not 80% as suggested by Figure 1) occurred in 1984 and over the 60-year time period it averaged 6%.  Of course, the figures from other countries are also subject to the time-dependent biases in fishing effort and reporting systems as mentioned for Peru above. Therefore, this comparison should be heavily caveated.**

We have doubled checked our numbers and are correct. It seems we were not clear enough. The 80% record contribution of Peru to Pacific landings in 1984 is limited to sharks and not all chondrichthyans. The Figure 1 mentions this. The numbers estimated by the Reviewer are correct as well if all chondrichthyans (sharks, rays and skates) are included **.**

**With regard to the discussion of trade, it would have been better to present annual values (perhaps in a table) rather than aggregated amounts for 1997-2012. The important point about Peru having picked up the trade in shark fins when Ecuador banned this trade in 2008 should have been illustrated in a table or figure. I wonder if the authors were able to explore how much of the Peruvian imports during this period were from fishing vessels choosing to land shark products in Peru versus from traders re-routing their consignments through Peru.**

We have presented in a table (per year) the import and export of Peruvian shark products

*.*

**What role do foreign fishing vessels play in Peruvian shark landings and has this changed over time?**

Hard to tell on how the data is reported.

**It would also have been interesting to hear about the trade in skate and ray wings, and whether this follows similar patterns as shark fins or is primarily for the domestic market.**

We don’t have that data as is not reported as such.

**In terms of management, the authors should be clear in recommending whether a maximum size or a minimum size is a better strategy--the discussion seems to make points on both sides of this argument. Also, what is the status of implementation and enforcement in Peruvian fisheries? Finally, since the authors claim that shark finning is not practiced because sharks are utilized for their meat, it is not clear why a shark finning ban is recommended as a management priority. It seems under current circumstances in Peru that recommending catch limits might be a more effective way of controlling shark mortality within sustainable limits.**

In the discussion section we included:

*"The regulation of the minimum capture length leaves open and unregulated the fishery for the adults which is detrimental for the population. The protection and management of a shark breeding area does not guarantee that the population is healthy in the absence of a lack of management for other size classes (Kinney et al., 2009). Demographic models and experiences in the shark fishery management indicate that focusing the management on adult would be more beneficial for the total population (Musick et al., 1999; Brewster-Geisz et al., 2000; Cortés et al., 2002; Gallucci et al., 2006). For example, over 30 years the management of Galeorhinus galeus fishery in South Australia was oriented toward protecting their breeding areas. However, populations declined until the fishery collapse. This was because the adults were fish unsustainably during those 30 years (Kinney et al., 2009). Taking this as experience, the Mustelus antarcticus fishery in southeast of Australia focused their effort on a single class, youth, resulting in a sustainable fishery (Prince et al., 1992). This way, the minimum capture length regulation should be reevaluated.*

*Third, the regulation is not implemented and enforced; foremost many of the fishermen are unaware of it. The minimum capture length for the mako shark is set at 170 cm but IMARPE reports that 60.1% (year 2009) and 88.5% (2010) of mako landings in the port of Pucusana were under minimum legal size (Anuario CientíficoTecnológico Vol. 5–10, 2005–2010). Of the 41 fishermen interviewed (in the landing points of Paita, Salaverry and Ilo), 93% do not discard any shark once it is hooked, only 34% reported that they discard sharks below 40-60 cm, and only 4.8% were aware of the regulations (Mangel et al., 2007)."*
